# Design Concepts and Performance Characterization of Heat Pipe Wick Structures by LPBF Additive Manufacturing

**DOI:** 10.3390/ma15248930

**Published:** 2022-12-14

**Authors:** Konstantin Kappe, Michael Bihler, Katharina Morawietz, Philipp P. C. Hügenell, Aron Pfaff, Klaus Hoschke

**Affiliations:** 1 Fraunhofer Institute for High-Speed Dynamics (EMI), Ernst-Zermelo-Str. 4, 79104 Freiburg, Germany; 2 Fraunhofer Institute for Solar Energy Systems (ISE), Heidenhofstraße 2, 79110 Freiburg, Germany

**Keywords:** additive manufacturing, heat pipes, laser powder bed fusion, wick structures, heat pipe performance

## Abstract

Additive manufacturing offers a wide range of possibilities for the design and optimization of lightweight and application-tailored structures. The great design freedom of the Laser Powder Bed Fusion (LPBF) manufacturing process enables new design and production concepts for heat pipes and their internal wick structures, using various metallic materials. This allows an increase in heat pipe performance and a direct integration into complex load-bearing structures. An important influencing factor on the heat pipe performance is the internal wick structures. The complex and filigree geometry of such structures is challenging in regards to providing high manufacturing quality at a small scale and varying orientations during the printing process. In this work, new wick concepts have been developed, where the design was either determined by the geometrical parameters, the process parameters, or their combination. The wick samples were additively manufactured with LPBF technology using the lightweight aluminum alloy Scalmalloy^®^. The influence of the process parameters, geometrical design, and printing direction was investigated by optical microscopy, and the characteristic wick performance parameters were determined by porosimetry and rate-of-rise measurements. They showed promising results for various novel wick concepts and indicated that additive manufacturing could be a powerful manufacturing method to further increase the performance and flexibility of heat pipes.

## 1. Introduction

The high energy and packing densities in certain industries, such as the automotive industry with its electrically powered cars, high-tech and computer hardware industries, and the aviation and space flight industries, drive the need for effective and reliable heat transfer. With this, heat pipes are gaining relevance as they have the ability to effectively transfer heat over a large distance [1]. The vital elements of the heat pipes are the inner wick structures [2,3]. They enable fluid transfer through capillary action and thus significantly determine their performance. However, the currently available uniform wick structures, such as grooved or sintered structures, suffer from conventional manufacturing constraints [4,5,6]. These limit their size and shape and thus the performance of the heat pipe [7,8]. Therefore, grooved and sintered shape type structures have recently been combined within a wick design in order to achieve better properties [7]. However, with conventional techniques, this requires increasingly complex manufacturing routes.

Current progress in the additive manufacturing of metals, in particular Laser Powder Bed Fusion (LPBF), offers a promising manufacturing process, as it allows great design freedom in terms of shape, geometry, and material properties [9,10]. These advances have created new possibilities for the design of heat-transferring devices [11,12]. Specifically, the additive manufacturing of heat pipes can enable new design possibilities and improved performance. New and unusual heat pipe geometries, as well as their direct integration into functional components, can be achieved [13,14,15,16]. Furthermore, the manufacturing conditions are customizable to a large degree, e.g., by adapting the laser exposure, such that the material properties of the wick structure could also be advanced and tailored on a small scale. This allows the inner wick structures to be directly determined by the geometric design [8,11,12,17] or the process parameters [18].

Ameli et al. [8] used LPBF with aluminum powder to manufacture cubic porous wick samples by forming octahedral unit cells with a regular and random distribution. These samples were characterized by their permeability and porosity and achieved comparable values to conventionally sintered wicks. Jafari et al. [19] used analysis methods introduced by Holley and Faghri [20] to conduct a detailed analysis of similar additively manufactured porous wicks from stainless steel and recorded significantly higher permeabilities and wick performance parameters at similar porosities. In further work, Jafari et al. [18] utilized the process parameters of the additive manufacturing process to create a porous bulk material using stainless steel suitable for heat pipe wick structures. They investigated the capillary performance, porosity, and pore radius of the printed samples, measuring an average porosity of 2.5–43% and pore radii of 9–23 µm. An application of an additively manufactured primary wick of a Loop Heat Pipe (LHP) was tested by Esarte et al. [6]. They developed geometrically determined porous structures and manufactured them using stainless steel powder. The heat transfer test showed a 10% increase compared to a LHP with a conventionally manufactured primary wick. Chang et al. [21] manufactured a flat aluminum heat pipe with grooved wick structures. The authors suggested that the rough surface of the additive manufacturing process further increases the capillary performance. In a previous case study, Kappe et al. designed and integrated various concepts of heat pipes into complex test structures [15]. The test samples were additively manufactured from Scalmalloy^®^ by LPBF and examined in thermal experiments. The comparison revealed multiple challenges of the different structural concepts and manufacturing characteristics. However, the general feasibility was demonstrated and the first promising integrated heat pipes could be fabricated.

While additive manufacturing has the potential to push the boundaries of possibilities for heat pipe design and has motivated a range of research work, it still has some challenges and limitations:

- Metals with low density and high thermal conductance, such as aluminum, are favored for heat pipes, as they enable a lightweight design and increase the heat dissipation over the structure [1]. However, in LPBF, the properties of aluminum alloys pose a challenge for manufacturing filigree wick structures as the resolution is typically decreased in comparison to other materials. Many studies, therefore, are still confined to low thermal conductance materials such as steel or titanium alloys.
- The characteristics of LPBF depend strongly on the direction of printing. The presented results and improved wick characteristics have mostly been based on samples manufactured in the ideal, vertical orientation, where no overhang is introduced by the cylindrical shape. For designs with a more complex shape or the integration into a structural component, the printing orientation can vary for each section of the heat pipe. With increasing deviation from the ideal position, multiple problems arise, such as the necessity of support structures and specialized laser exposure strategies such as downskin.
- The characterization of the wick performance differs depending on the test method used, which makes comparability between different studies using additive manufacturing and conventional designs difficult.

This work aimed to develop different concepts for additively manufactured wick structures to achieve better wick performance and manufacturability even with unfavorable printing directions and materials. Different characterization methods were used to investigate these influences. The wick concepts presented in this paper were designed for an exemplary application in the optical bench of a nanosatellite [22,23,24]. However, the design and integration of the heat pipe still require further investigation. Grooved wick structures with different geometries, porosities, and hybrid concepts were designed and manufactured by LPBF using Scalmalloy^®^ metallic powder. In addition, the influence of the manufacturing parameters and the printing orientation was investigated. The concepts were examined by means of optical microscopy to evaluate the manufacturing quality. With mercury intrusion porosimetry and helium pycnometry combined with rate-of-rise measurements, the wick performance of the manufactured samples was characterized. The results of the different measurement methods were compared to identify deviations and possible limits of the setups.

## 2. Design of the Wick Samples

The different concepts for additively manufactured wick structures can be classified into three different categories, whereby the design of the wick structure is determined by

- its geometric parameters,
- the LPBF process parameters,
- both the geometric parameters and process parameters.

**Figure 1:**
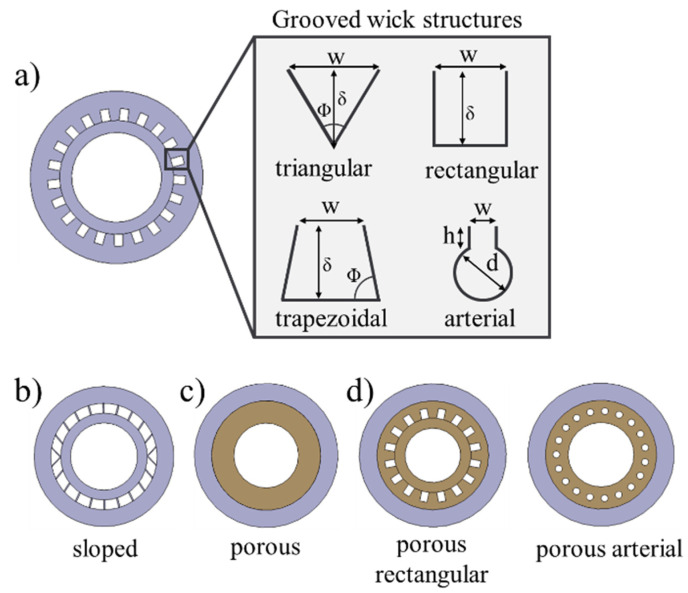
Different wick structures: (**a**) geometrically determined grooved wick structures and characteristic dimensions, (**b**) sloped grooved wick structure, (**c**) porous wick structure, and (**d**) hybrid wick structures.

**Figure 2:**
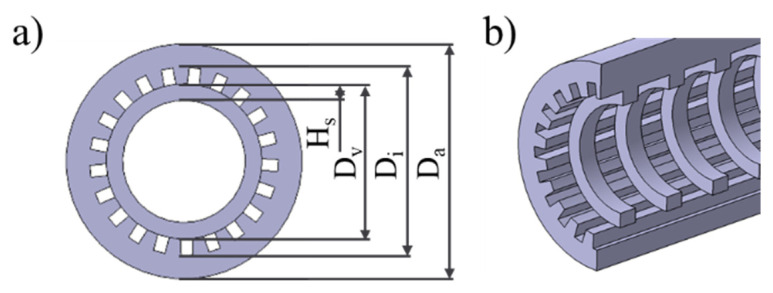
(**a**) Characteristic dimensions of wick sample and (**b**) support rings inside the grooved wick structures.

For the geometrically defined wick structures, different groove shapes, namely triangular, rectangular, trapezoidal, arterial, and sloped grooves were selected. Figure 1a shows the CAD models of the grooved wicks and their characteristic geometrical parameters. A parameter study was carried out to provide initial statements on the required characteristic dimensions. This was based on a detailed theory described in [25], which refers to a one-dimensional model. An optimization for the respective case was conducted by calculating various parameter combinations. The characteristic dimensions, as seen in Figure 1a and Figure 2a, were outputs of the parameter study and are summarized in Table 1. The sloped grooves shown in Figure 1b were developed to compensate for some of the problems arising from a horizontal printing orientation. The walls between the groove had a varying angle to the horizontal direction, ranging from 90° at the bottom to 45° in the middle. In this way, overhanging surfaces with angles under 45° were avoided. At the same time, the walls were designed to be fabricated with a single laser track each to minimize unwanted fusing of unmelted powder.

The geometry of the porous wick structure shown in Figure 1c was defined by the LPBF process parameters and consisted of a cylindrical volume element inside the solid outer wall. At first, the pores were deliberately formed by the manufacturing process. For the fabrication of porous structures, three main parameters were identified from previous experience and parameter studies: the laser power P, the laser track scan speed v, and the hatch distance h between the laser tracks. These parameters were varied to produce different porous samples. Multiple approaches exist for obtaining a single parameter to compare the processing conditions; see [26]. Here, the specific energy density e was used, which is defined as:

(1) $$e=P/\left(v\times h\right)$$

In order to create a fluid loop in the heat pipe, a porosity ε of approximately 45%, comparable to conventionally sintered wicks, and a permeability K > 1 × 10^−10^ were determined by the parameter study.

The porous rectangular and porous arterial wick as shown in Figure 1d aimed to increase the wick permeability by implementing paths with low flow resistance into the wick. At the same time, the porous sections should lead to a high capillary force. As there are no theoretical models to predict their performance, the same manufacturing parameters were applied and the same porosity values were targeted.

The intended minimum wall thickness of t ≥ 1.0 mm was recommended in the material data sheet [27] and the guide value of overhang angles δ ≤ 45° could not be met for all designs and printing orientations. To test the boundaries of manufacturability for structures deviating from these guidelines, non-standard manufacturing parameters were used based on experiences from previous parameter studies. Furthermore, inside the vapor channel of the grooved and hybrid wicks, support rings were implemented, as can be seen in Figure 2b. They supported the upper section of the wick when printing horizontally to reduce related problems in manufacturability. The depth of the rings was 1.0 mm with a height $H_{s}$ of 0.7 mm, spaced out with gaps of 2 mm along the samples. This resulted in a 33% coverage of the liquid-vapor interface in the vapor channel, which might have negatively influenced the fluid loop but increased the manufacturability.

## 3. Additive Manufacturing by LPBF and Specimen Preparation

The wick concepts were additively manufactured using an EOS M 400 LBPF machine. The specimens were made of the aluminum alloy Scalmalloy^®^ with a particle size distribution of D10 = 2010 μm, D50 = 3610 μm, and D90 = 5373 μm measured with a Microtrac MRB Camsizer X2. A layer thickness of 60 μm for all samples was specified. To provide an inert atmosphere, nitrogen was used. A rotating stripe scanning strategy with scan vectors rotated for every layer was utilized. In addition, a single contour exposure was used for the geometrically determined wick structures, while no contour exposure was used for the porous samples. For the manufacturing of the grooved wick concepts, five different manufacturing contour parameter sets were chosen, as shown in Table 2. The contour laser track parameter draws the outline and therefore has the biggest influence on the surface quality and the resolution of surface features. Parameters P1 and P2 were process contour parameters commonly used for printing bulk material of Scalmalloy^®^ at Fraunhofer EMI, whereby parameters 3–5 were adapted for the printing of the filigree grooves.

The parameters for the porous wick samples were chosen based on the results from preliminary porosity measurements. The influence of the energy density and hatch was visible in the density of the wicks. The porosities determined with the weighing method ranged between 38% and 60% and therefore achieved the target porosity of 30% to 60%. Here, the main influence was the energy density, with the lowest energy density reaching the highest porosity. Simultaneously, the porosity decreased with the hatch distance. Three different energy densities with different laser power and relatively high hatch distances between 0.46 and 0.67 mm were picked based on two criteria: high porosity and small pores. The porosity of the sample should be high to increase the permeability of the wick. Simultaneously, the pores should be small to increase the capillary pressure as the driving force of the fluid loop. The porous wicks were printed using the parameters shown in Table 3.

The removal of the residual powder after the printing process was done in two steps. By cleaning with compressed air, the majority of the remaining powder was removed. In a final cleaning step, the samples were dispersed in an ethanol bath and treated in an ultrasonic bath. This way, the residual powder, especially in the fine pores of the porous wicks, could be further reduced. However, single powder residues could still be detected. For all samples, a specimen of 10 mm in length for the optical microscopy was cut with an abrasive wet cutting machine. This cutting process created a planar surface on the wick sample, which was used as a contact point with the liquid in the rate-of-rise measurements. For the samples examined with the mercury intrusion method, two additional 10 mm specimens were prepared.

The specimens for the optical microscopy were further processed. Firstly, they were cold-mounted in epoxy resin. Then they were ground and subsequently polished. Polishing cloths with different grain sizes were used to increase the surface quality. This process was cooled with a lubricant and a diamond spray was used as a polishing agent.

## 4. Experimental Setup

### 4.1. Microscopy and Preliminary Measurements

**Figure 3:**
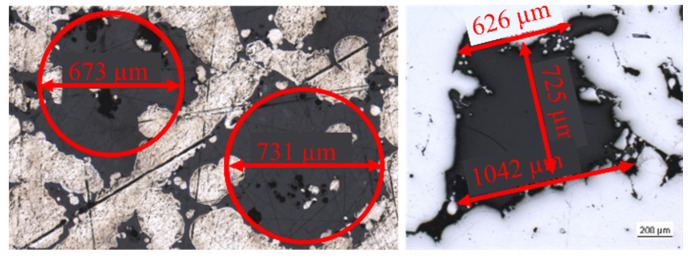
Exemplary measurements of pores (**left**) and trapezoidal grooves (**right**).

To study the microstructure of the wick samples, a Zeiss Axio Imager.Z2m optical microscope was used. The microscope was equipped with a camera to produce digital images. The microstructure features, such as the characteristic dimensions of the grooved wicks and the pore diameter of the porous wicks, were measured, as seen Figure 3.

For a preliminary assessment of the porosity of the porous wicks, cubical samples were manufactured with identical process parameters. Their porosity was determined by the following relation [28]:

(2) $$\varepsilon =1-\frac{\rho_{a}}{\rho_{t}}$$

where $\rho_{t}$ was the bulk material density. The apparent density was $\rho_{a}=m/V$, where the mass m was determined by weighing and the volume V by measuring the outer dimensions of the cube samples.

### 4.2. Mercury Intrusion Porosimetry

The mercury intrusion porosimetry was carried out with a Quantachrome Poremaster 60. It measures pore sizes between 3.6 nm and 1100 μm by intruding the sample with non-wetting mercury [29]. The evaluated sample was placed in a glass cell. The latter was connected to a pressure chamber and evacuated. With the following intrusion of mercury, the pressure increased continuously. Simultaneously, the volume change was recorded at the stem of the measurement assembly. This dataset was then further analyzed by applying the Washburn equation, first formulated in [29,30]:

(3) $$p=\frac{-2\sigma *\cos\theta }{r_{p}}$$

wherein σ represented the surface tension of the liquid, θ the contact angle between the liquid and the solid, which was assumed to be 140° for the combination of mercury and aluminum, and $r_{p}$ as the cylindrical pore radius. By differentiation of (3) with the assumption of constancy of σ and θ, the volume pore size distribution $D_{v}\left(r_{p}\right)$ was determined to be:

(4) $$D_{v}\left(r_{p}\right)=\frac{p}{r_{p}}\frac{dV}{dp}$$

A series of ΔV/Δp measured with the mercury intrusion method as a cumulative curve was then reduced to a distribution of pore volume per radius interval. Furthermore, the permeability of the wick structure could be determined as a function of the pore diameters and porosity [31]:

(5) $$K=\frac{\varepsilon_{wick}d_{p}{}^{2}}{16\tau }$$

with $d_{p}$ being the mean pore diameter gained from analyzing the pore size distribution. The porosity was calculated to be

(6) $$\varepsilon_{wick}=V_{intruded}/\left(A_{wick}*l\right)$$

with $V_{intruded}$ being the total volume of the intruded mercury and $A_{wick}$ the wick area of the sample. The measured porous area in the polished cut image was defined as the wick area and l was the sample length. The effective pore tortuosity τ was a modeled measure for the deviation of the pore shape of the straight cylindrical capillaries and straight diffusion paths. This was also determined in the mercury intrusion measurement as described in [32].

### 4.3. Helium Pycnometry

The Quantachrome MICRO-ULTRAPYC 1200e was used for further analysis of the density of the printed samples. A helium pycnometer was used to determine the true density of porous samples with open pores. This was based on the constant volume principle and included a sample and a reference chamber with a known volume *V_R_*, which was connected via an initially closed transfer valve. The sample was placed in the sample chamber with the volume *V_C_*, which was then filled with helium by increasing the pressure. The ultimate pressure $p_{1}$ was recorded and the transfer valve to the reference chamber opened. After the pressure equalization, the pressure $p_{2}$ was recorded. With these values, the sample volume $V_{P}$ could be calculated by [33]:

(7) $$V_{P}=V_{c}+\frac{V_{R}}{1-\left(p_{1}/p_{2}\right)}$$

With the sample mass m and the apparent density $\rho_{a}$, the true density $\rho_{t}=m/V_{P}$ was put into $\varepsilon_{\mathrm{th}}=1-\frac{\rho_{a}}{\rho_{t}}$ to receive the theoretical porosity $\varepsilon_{\mathrm{th}}$.

### 4.4. Rate-of-Rise Experiment

**Figure 4:**
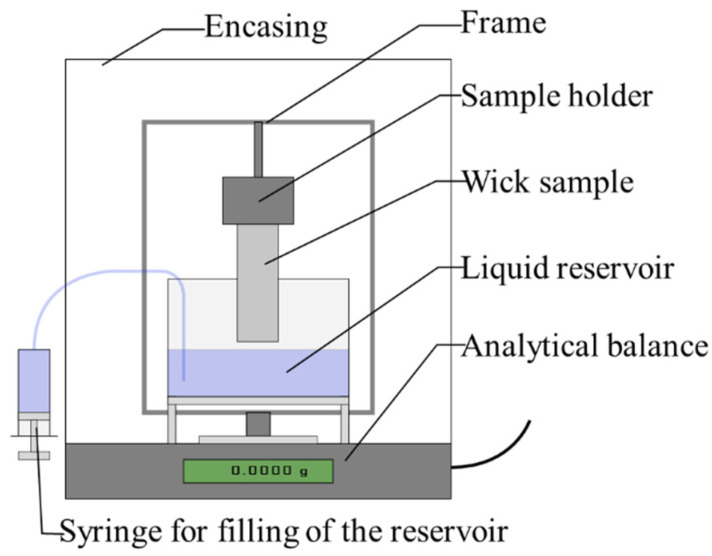
Rate-of-rise measurement setup.

For the rate-of-rise experiment, the Sartorius R160P analytical balance was used. For the experimental setup, a beaker with a platform, a frame to hang the sample, and a thermometer were used, as seen in Figure 4. The wick sample was hung into the frame with a sample holder. The latter had an opening with decreasing diameter in which to wedge the wick sample. By encasing the whole setup, the measurement error of the scale by circulating air, and the vaporization when using volatile working fluids were reduced. The temperature of the liquid and the ambient temperature were recorded with the thermometer to derive the fluid properties. Before each experiment, the balance was calibrated with an internal calibration weight.

As the working liquid, distilled water at 22 °C was used for the majority of the rate-of-rise experiments. It should be noted that distilled water is not the desired working liquid for the heat pipe application. It has been found that acetone in combination with Scalmalloy^®^ is a more viable working liquid. To bring the wick sample in contact with the liquid, a syringe was used to fill the reservoir. The filling was stopped at the moment of contact, which was observed when the liquid meniscus enclosed the bottom of the sample. The experiment was stopped after 60 s, as the equilibrium height was reached for all samples after this period. The output of the rate-of-rise measurements was the capillary pumping mass as a function of time. To derive the wick performance from this data, a linear fitting approach as commonly used in studies [7,19,34], was utilized. With the following assumptions, the liquid rising process can be described [20] as:

- One-dimensional and steady-state laminar flow in the wick,
- uniform saturation with liquid along the wetted length,
- no initial and entry effects in the liquid reservoir,
- and evaporation of the liquid is neglected as the closed space by the glass cover minimizes the evaporation of the test liquid.

For the rise of a liquid in the wick structure, the momentum balance of the capillary pressure $\Delta p_{cap}$, the pressure loss according to friction $\Delta p_{f}$, and the hydrostatic pressure $\Delta p_{h}$ in the wetted height of the sample was:

(8) $$\Delta p_{cap}=\Delta p_{f}+\Delta p_{h}$$

The description of the capillary pressure by the Laplace–Young equation, which is directly related to the Washburn equation [34] and the viscous friction by Darcy’s law, led to:

(9) $$\frac{2\sigma }{r_{eff}}=\frac{\mu \varepsilon }{K}h\frac{dh}{dt}+\rho gh$$

Here $r_{eff}$ was the effective pore radius with $r_{eff}=r_{p}/\cos\theta$, μ the dynamic viscosity, g was the gravitational acceleration, h the capillary rise height, and dh/dt the capillary rise velocity. For the porosity ε, the measured values from the helium pycnometry were used where available. A linear fitting method was used to gain the performance parameter $K/r_{eff}$. For that, Equation (9) was rewritten with the performance parameter $\Delta p_{cap}*K$:

(10) $$\Delta p_{cap}*K*\frac{1}{h}-\rho gK=\mu \varepsilon \frac{dh}{dt}$$

By defining x=1/h and y=dh/dt, the following equation resulted [34]:

(11) $$y=\underset{slope}{\underset{︸}{\frac{\Delta p_{cap}*K}{\mu \varepsilon }}}*x-\underset{intercept}{\underset{︸}{\frac{\rho gK}{\mu \varepsilon }}}$$

For this equation, a linear fitting could be performed with sets of data of x and y from the rate-of-rise measurements. Since the experiment measured the capillary pumping mass instead of the liquid height, the following relation was used [19]:

(12) $$h=\frac{m}{\rho \varepsilon A}$$

This assumed a constant porosity and cross-section of the wick A along the rise direction of the liquid. The parameters x and y were therefore obtained by:

(13) $$x=\frac{1}{h}=\frac{\rho \varepsilon A}{m}\;;\;\;\;\;y=\frac{dh}{dt}=\frac{1}{\rho \varepsilon A}\frac{dm}{dt}$$

The slope of this fitting line then could then be used to determine the wick performance $\Delta p_{cap}*K$ or $K/r_{eff}$. For the fitting, x and y-values were taken mostly from the intermediate rising period. At the later stage, the equilibrium height was reached and the values for y became very small.

## 5. Results and Discussion

Several experimental setups were carried out to evaluate the characteristics of the new wick concepts. For the grooved and porous wick structures, the following investigations were performed:

- Microscopy to evaluate process parameters and contour accuracy.
- Helium pycnometry to calculate the porosity.
- Rate-of-rise experiment to calculate the capillary performance.

Additionally, the porous wick structures were examined by mercury intrusion porosimetry to determine the open pore porosity and the wick performance.

### 5.1. Additive Manufacturing and Geometrical Analysis

In the following, the manufactured samples were presented using optical microscopy images. The geometries were measured, including the porosities of the porous samples.

#### 5.1.1. Grooved Wick Concepts

**Figure 5:**
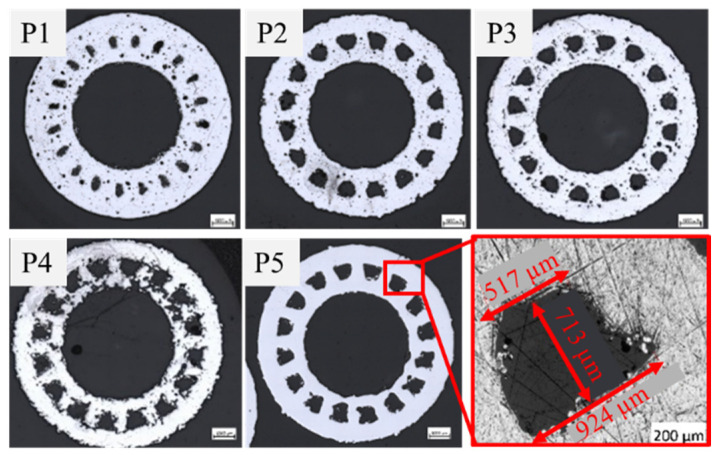
Optical microscopy images of vertically printed trapezoidal grooved wicks with manufacturing parameters P1 to P5.

One wick sample was manufactured for each groove type with each parameter, as shown in Table 2. The polished cut images of the vertically printed trapezoidal grooved wick samples are displayed in Figure 5. Sample 1, which was manufactured with parameter P1, showed the least conformity with the input geometry. Because of unwanted melting, the resulting grooves were narrow. Samples 2 and 3 had well-developed grooves, but the trapezoidal shape was not completely realized. The sample manufactured with parameter P4 showed good geometrical agreement but with a very rough surface due to the low energy density of the laser tracks. While the rough surface might prove beneficial for its function as a wick, the increased porosity of the outer hull could cause leakage problems. Sample 5 displayed the best results regarding groove shape and surface quality. Measurements of the characteristic dimensions, groove depth, and width, deviated less than 5% from the CAD model. In summary, while it was possible to produce acceptable results by choosing a standard parameter, adapting the process parameters of the contour and edge laser tracks positively affected the shape of the microstructure. With a very low power input, rough and partly porous groove structures could be achieved.

**Figure 6:**
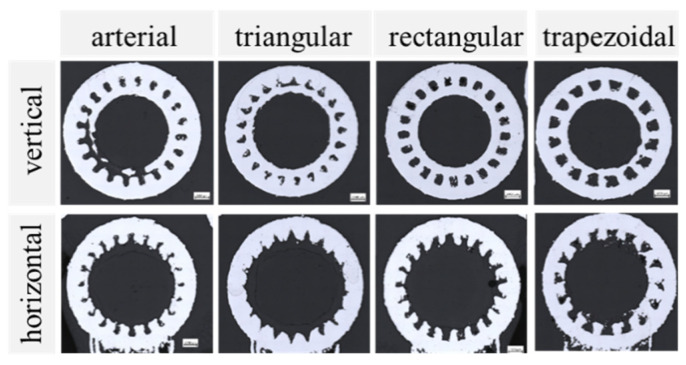
Optical microscopy images of grooved wicks printed vertically (**top row**) and horizontally (**bottom row**) with parameter P5 (P_Contour_ = 300 W; v_Contour_ = 500 mm/s).

In Figure 6, an overview of the four different grooved wick geometries, all printed with parameter P5, is illustrated. The diameter of the artery in the vertically printed arterial wick was represented well, while the entry channel was too wide. As the channel entry, being the liquid-vapor interface, is important for the function of the heat pipe, this area should be improved. The characteristic dimensions measured for the triangular grooves matched well with the input model. Unwanted melted powder partly blocked some of the grooves, which could hinder the liquid flow in the wick and therefore reduce the heat pipe performance. The rectangular and trapezoidal grooves both showed good results regarding geometric accuracy.

**Figure 7:**
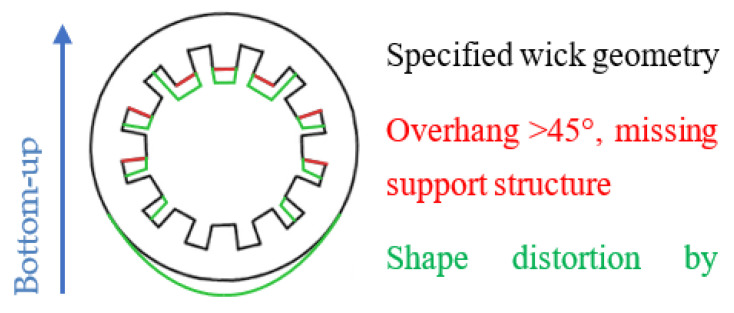
Problems that occur when horizontally printing grooved wick structures.

For the horizontally printed wick samples, the deviations from the intended shape were higher. The causes are summarized in Figure 7. The grooves at the top of the samples were elongated, while the grooves on the sides were deformed or missing completely. This was an effect of the penetration depth of the laser, which could lead to the unwanted melting of previous layers. Some areas had an overhang angle of over 45° and were only partially supported by the support rings. Increasing the amount of support further would decrease the area of the liquid-vapor interface, which would negatively impact the heat pipe performance. At the same time, it would increase the difficulty of removing the remaining powder from the grooves after the printing process.

#### 5.1.2. Porous Wick Concepts

**Figure 8:**
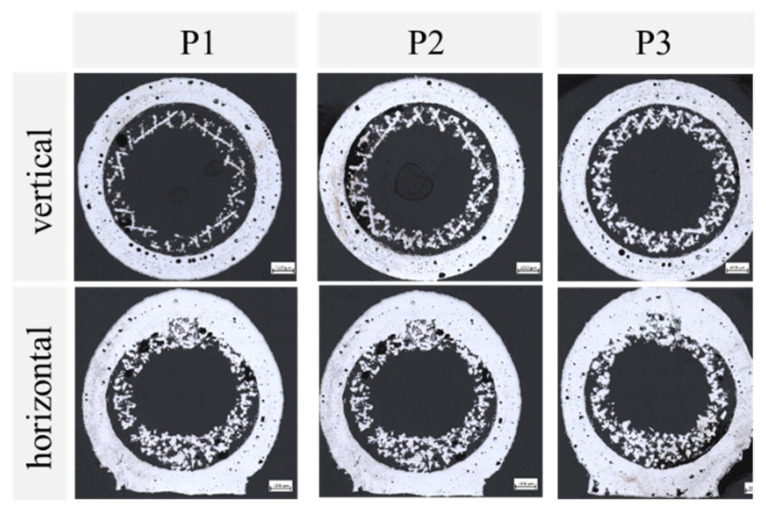
Optical microscopy images of porous wicks printed vertically (**top row**) and horizontally (**bottom row**) with porous process parameters P1 to P3.

The polished cut images of the produced porous wick samples, printed with parameters P1 to P3, are shown in Table 3 above and depicted in Figure 8. The vertically printed samples showed a separation of the porous wick in the center and the solid outer wall. The dimensions of the wall showed very small deviations compared to the CAD model input of less than 3%. The visible small pores could influence the leak tightness. The wick had a reduced thickness of up to 45% compared to the CAD model, which led to a gap between the inner wall and wick with an average size between 0.21 and 0.27 mm. The reduced volume of the wick had a direct impact on the achievable heat pipe performance. At the same time, the gap could have positively affected the fluid loop by increasing the overall permeability of the wick. Some designs, such as the annular heat pipe, make use of this effect [25]. This influence was less evident for the horizontally printed samples. They also showed a gap in the lower and side areas, while the top part was connected to the outer wall. The porous structure was densified in this area, as the laser melted the subjacent porous layers when it exposed the top of the outer wall.

**Figure 9:**
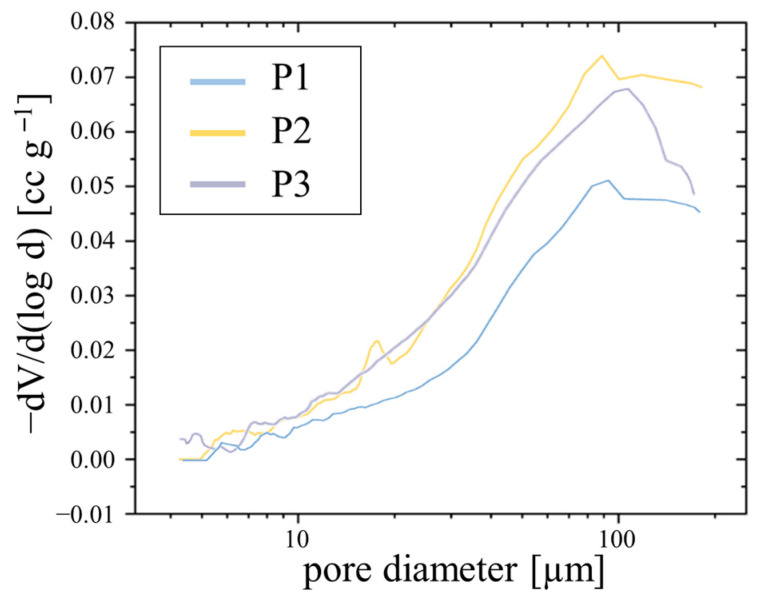
Pore size distribution of the vertically printed porous wick samples P1, P2, and P3.

A more detailed analysis of the pores was possible with the pore size distribution measured with the mercury intrusion method. The distributions of the horizontally printed porous wick samples are shown in Figure 9. This shows the intruded mercury volume over the pore diameter, normalized on 1 g of the sample. The depicted pores ranged from 3 to 200 μm and were sown in a logarithmic scale.

The graph shows a similar distribution, with the most prevalent pore diameter lying between 88 μm for P2 and 104 μm for P3. The average pore size for all samples was around 45 μm. These pore sizes were relatively small compared to the defined porous SLM structures presented in the literature [8,19]. Small pores benefit the achievable capillary pressure, while in combination with a low porosity also lead to a decrease in wick permeability. The horizontally printed porous samples P1H and P2H showed similar results in the measurements with a most prevalent pore diameter of around 108 μm.

**Figure 10:**
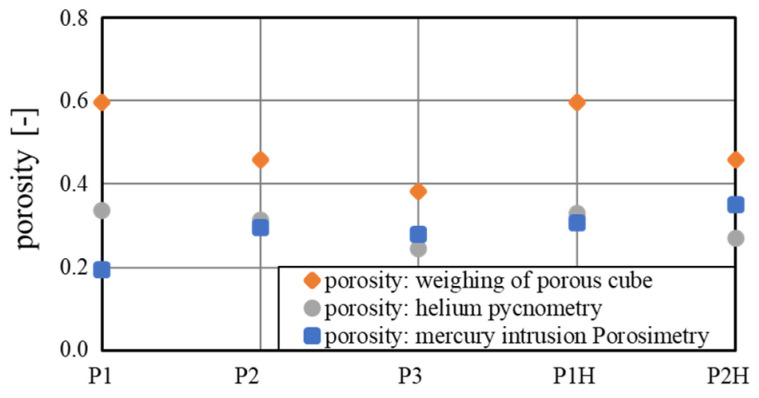
Measured porosities of porous wick concepts by weighting of a porous cube, helium pycnometry, and mercury intrusion porosimetry.

Figure 10 gives an overview of the measured wick porosities: the expected porosity from the preliminary measurements, the theoretical porosity from the helium pycnometry, and the porosity measured from the mercury intrusion. The porosities of the helium pycnometry and mercury intrusion agreed very well and ranged between 20% and 35%, as seen in Figure 10. For samples P1 and P2H, there was a larger deviation, which could stem from pores with a complex intrusion path. These could have been blocked to some extent for the liquid mercury, but accessible for the helium with low viscosity. Both measurements showed a discrepancy of up to 40% from the expected values determined in the preliminary measurements. The reason for this is most likely the limitations of the preliminary analyzation method, which included closed pores when measuring the apparent density. The two setups discussed in this chapter only included open pores, which were reachable by the fluid. Another reason could be the difference in geometry of the porous cube and the wick sample, which might have affected the microstructure and porosity. The trends of the expected porosity and the He-experiment corresponded well with the highest porosity achieved with parameter P1. The effect of vertical and horizontal printing orientation on the porosity was small considering the results from the He-measurement.

#### 5.1.3. Alternative Wick Concepts

**Figure 11:**
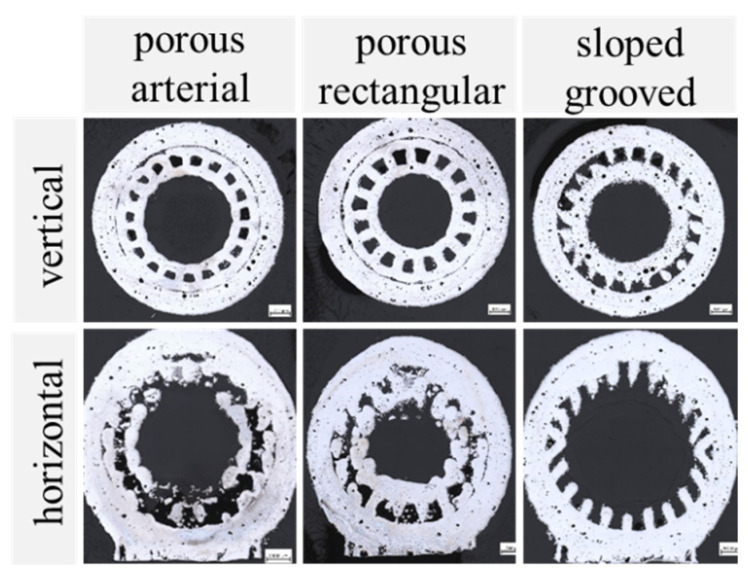
Alternative wick concepts printed vertically (**top row**) and horizontally (**bottom row**).

The benefit of the high design freedom of AM was used in the alternative concepts for wick structures. By adding artery and grooves, the porous wick took advantage of their increased permeability and enlarged liquid-vapor-interface, respectively. Figure 11 shows the alternative wick samples printed with parameter P1 (also see Table 3 above). Due to manufacturing restrictions, the results showed no porous structure in these wick samples. Even though the energy density in the wick was reduced in the same way as in the full porous structures, the small dimensions led to a complete melting of the contour tracks. The increased hatch distance could take no effect here, as the contour track, which resolved the groove/artery geometry, dominated. This was also true for the horizontally printed samples. A solution would be to increase the dimensions of the heat pipe or use a metallic powder with a lesser heat conductance. In this way, the geometrical resolution could improve, as the melting pool of the laser tracks becomes smaller.

More promising for an application with the given material and geometrical restrictions is the sloped grooves concept also shown in Figure 11. This showed well-defined grooves both for the vertical and horizontal printing orientation.

### 5.2. Characterization of the Wick Performance

The performance of the grooved wick concepts was characterized using the rate-of-rise method. For the porous concepts, the helium pycnometry and mercury intrusion experiments were additionally used and compared to the rate-of-rise measurements in Section 5.3.

#### 5.2.1. Grooved Wick Concepts

**Figure 12:**
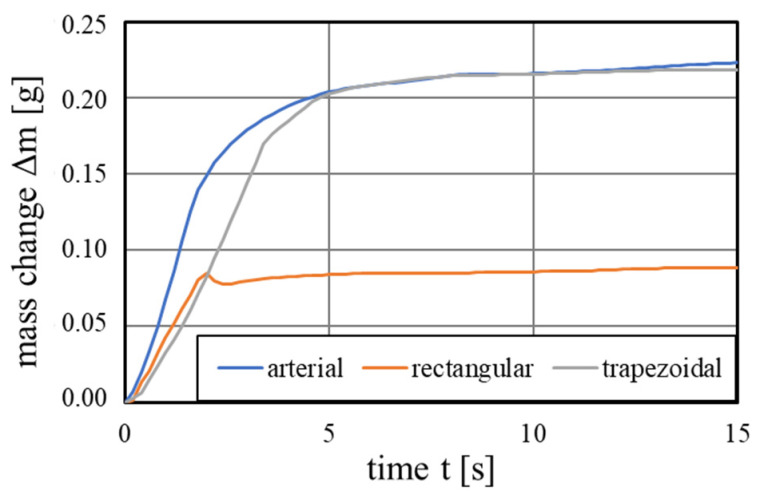
Capillary pumping mass of the vertically printed grooved wicks with the printing parameter 5.

Figure 12 depicts the recorded mass change as a function of time for the grooved wick concepts printed vertically with parameter P5. The triangular grooved wick is not shown, as no capillary pumping could be observed with these samples. The arterial and trapezoidal grooves achieved a similar equilibrium pumping mass, with the arterial groove having a steeper initial rising mass flow. The main rising occurred in the first 5 s, after reaching the equilibrium pumping mass, the mass flow strongly declined.

From these mass-time graphs, the following performance parameters were obtained by the previously described linear fitting method from Equations (11)–(13). The arterial and trapezoidal achieved similar performance parameters $K/r_{\mathrm{eff}}$ of 1.73 and 1.70 μm, while the rectangular wick achieved 0.28 μm.

**Figure 13:**
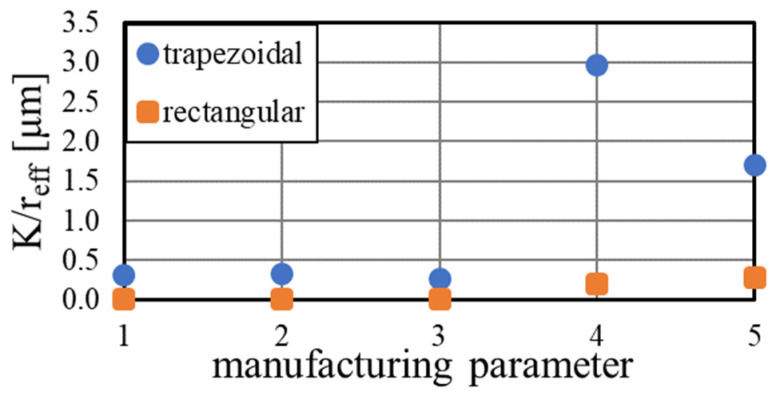
Performance of the trapezoidal and rectangular grooved wick dependent on the manufacturing parameter, calculated by rate-of-rise experiments.

The effect of the manufacturing parameter on the grooved wick performance is depicted in Figure 13. While the standard parameters resulted in a limited performance, parameters P4 and P5 showed a vast improvement. While the samples with parameter P5 showed the best match with the input geometry in the polished cut images, the rougher surface of sample P4 appeared to have a positive impact on the capillary pumping. The trapezoidal grooves 4 and 5 achieved a performance parameter of 2.96 and 1.70 μm, respectively. The rectangular grooves also showed the best performance manufactured with these parameters, but were significantly smaller. It appears that the angled channel entry and the higher groove volume of the trapezoidal grooves had a large impact on the wick performance.

#### 5.2.2. Porous Wick Concepts

**Figure 14:**
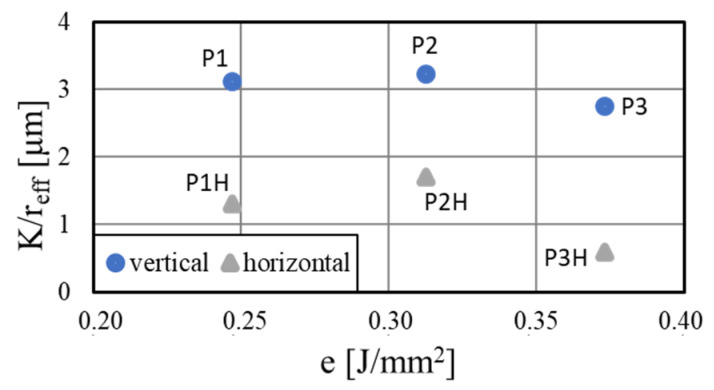
Performance of the porous wicks for different printing parameters and orientations calculated by rate-of-rise experiment.

Regarding the heat pipe performance, the lower porosity reduced the expected wick permeability. The achieved porosities were in the range of conventionally sintered heat pipes [35] and therefore could have led to a similar performance. The resulting performance parameters are shown in Figure 14. The performance $K/r_{\mathrm{eff}}$ of up to 3.22 μm for sample P2 was the same order of magnitude as a comparable study [19], which achieved values between 1.04 and 7.14 μm. The performance of the horizontally printed samples here was around a factor 2 lower than the vertically printed ones, indicating a strong influence of the printing orientation on the permeability of the porous wicks.

#### 5.2.3. Alternative Wick Concepts

Finally, the results for the alternative wicks are summarized in Table 4. The porous grooved wick achieved a performance similar to the regular grooved samples, while showing no pumping for the horizontally printed sample. Due to the very small arteries, the porous arterial wick had a very low performance but achieved a better result for the horizontal orientation. As seen in the polished cut image in Figure 11, the wick showed slightly porous parts and a groove-like structure, which could explain the comparably good performance result. The sloped grooves accomplished satisfying values, especially in the first revision. A high groove volume, as is present in these samples, apparently had a positive influence on the resulting performance.

### 5.3. Comparison of the Measurement Methods

**Figure 15:**
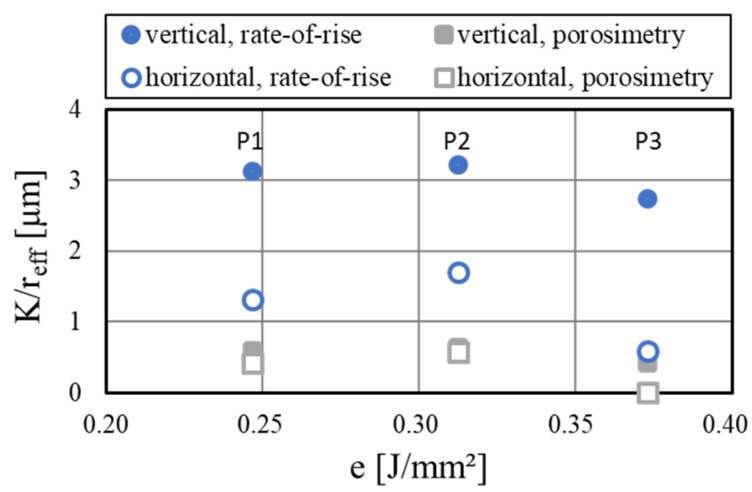
Comparison of the porosimetry and rate-of-rise measurements for the porous wick samples.

The two investigation methods presented in this paper, the porosimetry by mercury intrusion and the rate-of-rise measurements, both enabled the characterization of the heat pipe performance with the parameter $K/r_{\mathrm{eff}}$. For the mercury intrusion porosimetry, the measured permeability values were divided by the effective pore radius $r_{eff}$. This radius was gained by $r_{eff}=r_{p}/cos\theta$, with the measured average pore radius *r_p_* and the contact angle of water-aluminum θ of 45° [25]. As is shown earlier, the rate-of-rise measurements provided $K/r_{\mathrm{eff}}$ by the linear fitting. The resulting values as a function of the specific energy density (P1: 0.25 J/mm²; P2: 0.31 J/mm²; P3: 0.37 J/mm²) are compared for the vertically and horizontally printed porous wick samples in Figure 15.

A large deviation of absolute values could be observed when comparing the two measurement methods, with the performance parameter gained from the porosimetry being considerably lower. Possible reasons are the differences in the data reduction approaches, which both rely on different assumptions. For the porosimetry, the permeability was modeled with cylindrical pores, while the pore radius was measured directly. In contrast, the rate-of-rise experiment was modeled with a one-dimensional and steady-state laminar flow in the wick and needed more external input values like fluid properties. While the absolute values differed, the trends of both measurement methods agreed very well. With both setups, peak performance at a medium energy density could be observed. The reduction of performance when printing in horizontal orientation could also be resolved with both methods but was more apparent in the rate-of-rise measurements. This was probably due to the influence of the flow direction, which was not considered in the porosimetry measurements. For the characterization of the performance of different wick concepts, both methods were applicable on their own, while the comparability of their absolute results was limited. At the same time, the comparison of the different concepts should not overly rely on the measured $K/r_{\mathrm{eff}}$ value. While it is a good indicator of performance for variations of the same concept, the different working principles of the concepts might not be properly be displayed by these measurements. To investigate the wick performance more closely, more complex test setups such as thermal conductance experiments are required.

## 6. Conclusions

In this work, new concepts for LPBF additively manufactured wick structures were investigated. Various designs were developed which exploited the design freedom of additive manufacturing. The wick structures were determined by geometric parameters, process parameters, or a combination of both. Furthermore, the influences of the printing direction on the wick structures were determined. For all the different test specimens, the characteristic capillary parameters porosity, permeability, effective pore radius, and capillary performance were determined and discussed. Thereby, the rate-of-rise experiment, helium pycnometry, and mercury intrusion porosimetry were applied and the influence on the results was identified. The main conclusions of this work are the following:

- Additive manufacturing with LPBF enables the manufacture of different wick concepts through geometric design, process parameters, or a combination of the two. The grooved wick concepts, especially with trapezoidal and arterial groove geometry, achieve very good wick performances $K/r_{\mathrm{eff}}$ of up to 3.0 μm. However, standard manufacturing parameters are not suitable for accurately reproducing the CAD design of filigree structures. It is necessary to develop special process parameters. Furthermore, the porous wick structures achieve a performance $K/r_{\mathrm{eff}}$ of 2.7 to 3.2 μm for the vertically printed samples. This demonstrates their great potential, but many closed pores are still suspected. Further adjustments to the process parameters are necessary.
- The printing direction has a significant effect on the printing quality of the wick structures and thus on the capillary performance. Specific process parameters, geometric features such as inner support rings, or adapted geometries such as the sloped grooves, also enable printing in the horizontal direction. Specifically, sloped grooves show an extraordinary performance parameter $K/r_{\mathrm{eff}}$ of 8.9 μm, which demonstrates the potential of this concept. This is especially promising when using heat pipes with complex profile shapes, where it is not always possible to manufacture in the optimum orientation.
- Different measurement methods for evaluating the wick performance show large deviations and are therefore only comparable with each other to a limited extent. Whereas measurements with the aid of porosimetry cannot take into account the flow direction of the liquid, the rate-of-rise experiment can, which is important for the comparison of the different print orientations. However, the experiments here showed a good initial comparison between the different concepts, but to enable an accurate evaluation of the wick concepts, thermal conductance and heat transfer limit experiments of filled heat pipes are essential.
- The production of filigree wick structures from metals with high thermal conductivity is challenging. The use of different aluminum alloys enables a higher printing resolution, which provides further optimization potential for the geometrically defined wick structures.

In summary, the presented study showed the great potential of additive manufacturing with LPBF of wick structures, which in the future could allow the heat pipes to be produced in a single manufacturing step. Furthermore, the wick performance, and thus the heat conduction of the heat pipes, can be further increased through adapted and optimized wick designs.

## Figures and Tables

**Table 1:** Grooved wick dimensions.

Groove Type	Dimension	Value
general	outer ⌀ D_o_ [mm]	10.0
	inner ⌀ D_i_ [mm]	8.0
	vapor channel ⌀ D_v_ [mm]	6.6
	groove height δ [mm]	0.7
	sample length L [mm]	50.0
triangular	groove width w [mm]	0.5
	opening angle Φ	39.3°
rectangular	groove width w [mm]	0.5
trapezoidal	groove width w [mm]	0.5
	opening angle Φ	70°
arterial	groove width w [mm]	0.25
	artery ⌀ d [mm]	0.5
	channel height H [mm]	0.25

**Table 2:** Contour parameters for grooved wicks, with parameters P1 and P2 as contour parameters commonly used for bulk material with different surface roughnesses, and parameters P3, P4, and P5 adapted for printing filigree structures.

Parameter	P_Contour_ [W]	v_Contour_ [mm/s]
P1	600	400
P2	900	3000
P3	600	3000
P4	100	250
P5	300	500

**Table 3:** Printing parameters for porous wicks.

Parameter	P1	P2	P3
P [W]	335	600	500
v [mm/s]	2950	3000	2000
h [mm]	0.46	0.64	0.67
e [J/mm^2^]	0.25	0.31	0.37
ε [%]	59.6	45.8	38.4

**Table 4:** Performance of the alternative wick concepts.

K/r_eff_ [μm]	Porous Grooved Wick	Porous Arterial Wick	Sloped Grooves	Sloped Grooves, 1st Rev.
vertical	1.450	0.017	1.971	8.943
horizontal	0.000	2.277	1.530	-

## Data Availability

The data and results involved in this study have been presented in detail in the paper.
